# Supplementary material for: Trends of Eurasian Perch (Perca fluviatilis) mtDNA ATP6 Region Genetic Diversity within the Hydro-Systems of the Eastern Part of the Baltic Sea in the Anthropocene
Source: Animals (Basel). 2023 Sep 29;13(19):3057. doi: 10.3390/ani13193057 (PMC10571732; doi:10.3390/ani13193057)
Supplement: Supplementary file 1 [file animals-13-03057-s001.zip › animals-2600043-Suplementary Material Document S1.pdf]

animals

Supplementary Material Document S1

1. Results

1.1. Additional Details about Haplotypes A, B, C, and D Distribution Patterns

We found haplotype A alone among all 11 perch samples (codes 1–11) (Table 1). We observed the greatest frequency of this haplotype in Lake Žeimenys (13/17 sequences, corresponding to 76.47%). Haplotype B dominated in the Belarussian samples (19/37 sequences corresponding to 51.35%; all 10 individuals had this haplotype in the Meleshkovichi River channel). Haplotype C was most prevalent in individuals from the Mozyr sample (11/23 sequences, corresponding to 47.83%) in Belarus but it was totally absent in the Curonian Lagoon and the Elektrėnai Reservoir samples (Supplementary Table S1). The one available perch from the Chernobyl area also revealed the presence of this haplotype. Interestingly, we did not detect haplotype D among the perch samples from Belarus, Ukraine, and Lakes Drūkšiai and Žeimenys in Lithuania.

1.2. Additional PCoA Results

If two samples from Belarus (the Meleshkovichi River channel and Mozyr) were selected instead of one, then the PCoA results suggest one additional cluster represented by the homogeneous sample from the Meleshkovichi River channel (Supplementary Figure S1).

1.3. Genetic Differentiation among Perch Samples

Interestingly, pairwise comparisons of the samples in groups I and II (Figure 3) showed low Φ_{ST} values and were not significant ($p > 0.05$). We found the lowest (-0.077) Φ_{ST} value by comparing the Curonian Lagoon and the Dotnuvėlė River (Akademijos Reservoir) samples, but this difference was not significant. The lowest significant Φ_{ST} value (0.097; $p = 0.05$) was between the Neris River and Lake Engure samples. Although we observed the highest Φ_{ST} value by comparing the Meleshkovichi River channel and Lake Žeimenys samples – and it was highly significant ($p < 0.001$) – the Meleshkovichi River channel sample only comprised individuals with the single haplotype B. In addition, all comparisons of this sample with other samples showed Φ_{ST} values that indicated highly significant genetic differentiation between pairs of samples.

1.4. Haplotypes and Haplogroups of mtDNA ATP6 and D-Loop Regions of the Same Specimens

In general, there is a trend for distinct *ATP6* region haplotypes in specimens that are known to have phylogenetically less related D-loop haplotypes. Nevertheless, some individuals attributed to the same *ATP6* haplotype, and even the same haplogroup, possessed haplotypes belonging to different D-loop haplogroups. For examples, specimens with D-loop region haplotype C had *ATP6* region haplotypes B, C, or D that represent the B, C, and D haplogroups, respectively. Interestingly, specimens from China with the D-loop haplotype C had a unique *ATP6* region haplotype that could not be attributed to the ascribed haplogroups (Figure 1). Consequently, we also ascribed composite haplotypes for the *ATP6* and D-loop regions (Supplementary Table S2).

1.5. Comparisons of Trends of Haplotypes and Haplogroups of Perch Within the Studied Macrogeographic Area using Different mtDNA Markers

Of the D-loop haplotypes, only C1 and C4 were not detected outside of Belarus (D-loop, n = 44, six different sequences, including two unique haplotypes; *ATP6*, n = 37, three different sequences and no unique haplotypes). We did not identify heteroplasmic length variants [61] – that is, sequences with 10+ bp insertions. We detected an equal number of

unique haplotypes (including heteroplasmic length variants of the D-loop) in Latvia (D-loop, n = 178, 25 different sequences, including 17 unique haplotypes; *ATP6*, n = 40, eight different sequences, including four unique haplotypes) and Lithuania (D-loop, n = 267, 25 different sequences, including 17 unique haplotypes; *ATP6*, n = 108, eight different sequences, including four unique haplotypes). Because there were fewer individuals and samples from Latvia compared with Lithuania, we speculate that there is a trend for more unique haplotypes in Latvia represented by both mtDNA regions. Interestingly, in Lithuania we detected only two heteroplasmic length variants compared with five in Latvia. Five D-loop haplotypes (B4, B10, E, E1, and F8) identified in these two Baltic countries were not heteroplasmic length variants but had 1 bp deletions compared with the reference sequence. Finally, we found less heterogeneous genetic variability of the *ATP6* region (only four haplogroups found in the studied macrogeographic area instead of eight haplogroups identified based on the D-loop marker) [35], despite similar trends of overall haplotype distribution patterns among perch populations found within and between countries indicated by both markers.

2. Discussion

2.1. Potentials of Different Molecular Markers

The number of available perch mtDNA genomes and applications in intraspecific comparisons has increased over the years. In the near future perch, population genomics studies could be a common practice (reviewed by Vasemägi et al. [46]). Even so, investigations into perch population genetics based on smaller mtDNA fragments would still be relevant as a highly reproducible and less expensive method. In addition, smaller mtDNA fragments as molecular markers also have advantages to address specific scientific questions. For example, two specimens from China (represented by GenBank accession numbers AP005995 and MZ461595) could be clearly distinguished between each other and individuals from other geographic regions by its near complete (16,152 bp) mtDNA genomic data but not by the D-loop in the example presented by Vasemägi et al. [46]. This shows the great power of mtDNA genome sequencing as a valuable tool for perch genetic authentication of geographic localization [55]. At the same time whole-genome sequencing could be considered too sensitive and may not be suitable in phylogeographic studies reflecting time scale processes, such as the last glaciation events [39,62,63]. It has been demonstrated numerous times that the perch mtDNA D-loop is one of the best molecular markers to study events associated with the last glaciation in the Europe [35,38,41,62,63] and sometimes it can even reveal differences among perch populations that differ in their ethology [56]. However, the D-loop is considered to be a selectively neutral molecular marker and its application to evaluate possible anthropogenic impacts on genetic diversity of perch has failed to provide a clear picture [75].

2.2. Mutations in Different mtDNA Regions

Interestingly, two specimens from China differed from each other by three point mutations based on comparison of almost the entire mtDNA genome [46] but still had the same *ATP6* region haplotype and the quite common (in Eurasia) haplotype C of the D-loop region [41]. There were more point mutations in the unique *ATP6* region haplotype from Lake Balaton compared with the haplotype detected in China (Table 4 and Supplementary Table S2). This difference could be explained by the hypothesis that individuals carrying haplotype M of the D-loop represent an older evolutionary line of perch distributed in southern Europe [41]. The determined haplotype based on 16,152 bp of mtDNA was distinct from all other haplotypes, as it differed by more than 90 mutational steps compared with the nearest haplotype [46].

2.3. Short Summarized Information Regarding Research of Perch Phylogeography in Baltic Lands

Nesbø et al. [41] intended to shed light on postglacial migrations of freshwater fish in Europe. Hence, they undertook a comprehensive study on perch population genetics by using the available appropriate molecular techniques. For many years, the phylogeographic relationships they found among perch populations based on direct mtDNA D-loop region sequencing provided invaluable basic information for subsequent freshwater fish studies. Similarly, Toomey et al. [38] performed a large-scale phylogeographic study that greatly enhanced our understanding of perch genetic variability based on the D-loop and other mtDNA regions (*cytb*, *16S* RNA, and *COI*) as well as DNA microsatellite analysis. They conducted the study by using expanded materials collected from Western Palearctic sites and revealed five major genetic groups: the European Plain, the Danube drainage system, the Balkans, west-northern Fennoscandia, and eastern Europe. There was greater genetic variability of perch populations distributed along the eastern diagonal. Notably, the results based on DNA microsatellites were generally in agreement with the findings of the studied concatenated mtDNA fragment, except more perch genetic groups could be ascribed to the distinct geographic regions using microsatellites. Both studies [38,41] lacked comprehensive information regarding the genetic diversity of perch in the eastern part of the Baltic Sea Region, as low number of samples represented Lithuanian, Latvian, and Belarusian territories. More than a decade later, data from that study and expanded research [75] were combined into a final comprehensive investigation [35] that revealed complex phylogeographic relationships of perch populations in the eastern part of the Baltic Sea Region. This information, alongside previous investigations based on the same molecular marker [41,56,62,63], comprises the background that has allowed focusing on more detailed genetic, phylogeographic, and postglacial migration research in various macrogeographic areas. Currently, perch genetic investigations using larger local samples ($n > 20$) are still lacking in Ukraine and the Balkans.

2.4. Additional Recommendations Regarding Future Research of Perch Phylogeography

To get the other necessary parts of the puzzle regarding the origin and the number of the former perch refugia – especially those that have had a pronounced impact on the formation of current population genetic structure of perch inhabiting area between the Baltic and Black seas – and to reconstruct the postglacial migrations in Europe, samples from all other presupposed refugia of this species should be investigated. When corresponding molecular data on other taxonomically related (Percidae family) and other abundant, unrelated fish species (for example, the Cyprinidae family) become available, important scientific gaps in freshwater fish phylogeography in Europe will be filled [20,36,39]. It is also advisable to conduct more comprehensive research using species-specific DNA microsatellites [71–74] and in turn to evaluate the phylogeographic data based on DNA microsatellites [38], focusing on the macrogeographic area between the Baltic and Black Seas, as the genetic variability of perch populations in Lithuania and Latvia has been studied only fragmentally using DNA microsatellites that are not specific to perch [25,53,65]. Such accumulated data could be used for phylogeographic evaluation of perch and would create a background to track ongoing microevolution, including genetic changes associated with various anthropogenic objects in this geographic region.
