# Supplementary material for: Trends of Eurasian Perch (Perca fluviatilis) mtDNA ATP6 Region Genetic Diversity within the Hydro-Systems of the Eastern Part of the Baltic Sea in the Anthropocene
Source: Animals (Basel). 2023 Sep 29;13(19):3057. doi: 10.3390/ani13193057 (PMC10571732; doi:10.3390/ani13193057)
Supplement: Supplementary file 1 [file animals-13-03057-s001.zip › animals-2600043-Suplementary Material_Tables 2023-09-29.pdf]

animals

MDPI

Supplementary Material

Table S1. Different haplotypes detected in studied perch samples.

Code	Location	Basin	Haplotypes
		**Latvian Samples**	
1	Lake Engure	Baltic Sea	**A** (8), **C** (2), **D** (8), **D2** (1)
2	Lake Cirīšu	Daugava River	**A** (14), **A2** (1), **B** (1), **B1** (1), **C** (2), **C1** (1), **D** (1)
		**Lithuanian Samples**	
3	Lake Drūkšiai	Daugava River	**A** (13), **B** (2), **C** (6), **C2** (1)
4	Lake Žeimenys	Žeimena River	**A** (13), **C** (4)
5	Siesartis River	Siesartis River-Šventoji River	**A** (5), **A1** (1), **A3** (1), **C** (3), **D** (1)
6	Dotnuvėlė River (Akademijos Reservoir)	Dotnuvėlė River	**A** (3), **C** (3), **D** (7), **D3** (1)
7	Curonian Lagoon	Baltic Sea	**A** (2), **D** (4)
8	Elektrėnai Reservoir	Strėva River	**A** (5), **D** (2), **D3** (1)
9a	Neris River (Baltalaukis)	Neris River	**A** (5), **B** (2), **C** (2), **D** (3)
9b	Neris River (Buivydžiai)	Neris River	**A** (10), **C** (6), **D** (2)
		**Belarusian Samples**	
10a	Meleshkovichi River channel	Pripyat River	**B** (10)
10b	Mozyr	Pripyat River	**A** (4), **B** (8), **C** (11)
10c	Lake Aleksino	Pripyat River	**B** (1), **C** (1)
10d	Berezina River	Berezina River-Dnieper River	**A** (1), **C** (1)
		**Ukrainian Samples**	
11a	Ukraine (Chernobyl area)	Pripyat River-Dnieper River	**C** (1)
11b	Ukraine	Desna River	**A** (1), **B** (3), **C** (2), **D1** (1)

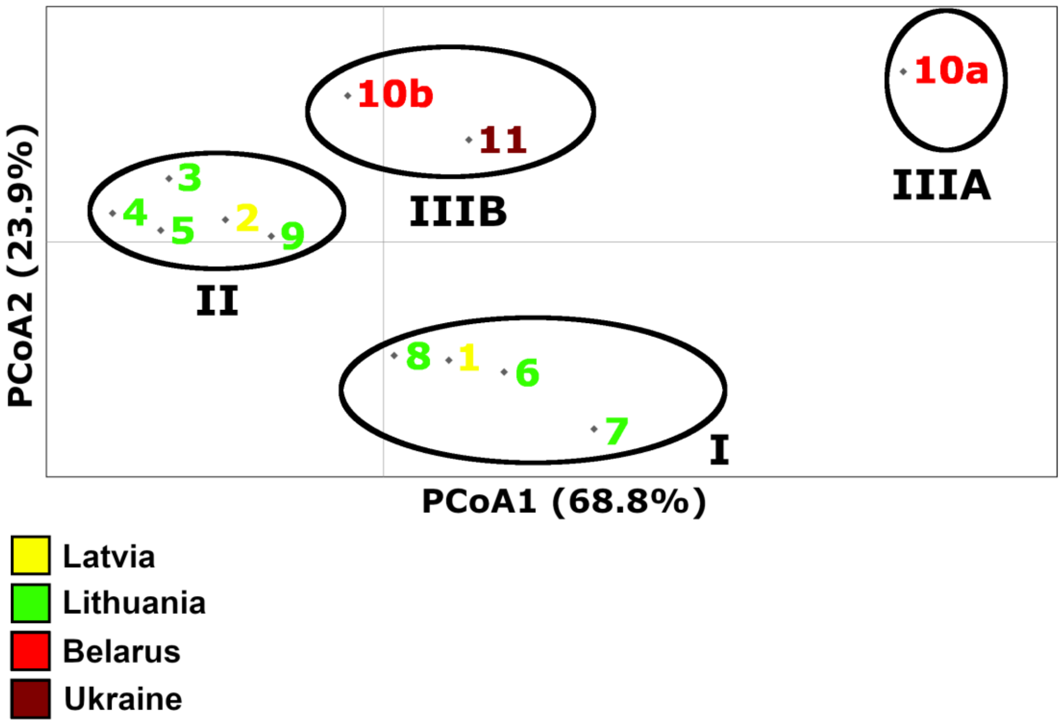

Figure S1. Principal coordinates analysis (PCoA) of Latvian, Lithuanian, Belarusian (comprised of only 10a and 10b samples), and Ukrainian perch samples. There are three genetic groups: I, II, and III. Codes: 1 – Lake Engure; 2 – Lake Cirīšu; 3 – Lake Drūkšiai; 4 – Lake Žeimenys; 5 – Siesartis River; 6 – Dotnuvėlė River; 7 – the Curonian Lagoon; 8 – Elektrėnai Reservoir; 9 – the Neris River; 10a – Meleshkovichi River channel; 10b – Mozyr; 11 – Ukraine.

Supplementary Table S2. Comparison of haplotypes and corresponding haplogroups at different mtDNA *ATP6* and D-loop regions using data of the same individuals from Cirīšu, Engure and Drūkšiai lakes [35], Chernobyl, Elektrėnai Reservoir and GenBank.

No	Individual Code	mtDNA Marker				
		ATP6 region		D-loop region		Composite *ATP6*-D-loop haplotypes
		Haplotype	Haplogroup	Haplotype	Haplogroup	
**Lake Cirīšu (n = 21)**						
1	C6	A	A	B4[a]	B	A-B4
2	C7	A	A	B	B	A-B
3	C8	B	B	J	J	B-J
4	C10	D	D	F7	F	D-F7
5	C11	A	A	B	B	A-B
6	C15	A	A	B	B	A-B
7	C17	A	A	B	B	A-B
8	C20	A	A	B	B	A-B
9	C22	A	A	B	B	A-B
10	C23	A	A	B	B	A-B
11	C25	C	C	C	C	C-C
12	C26	A	A	B	B	A-B
13	C28	A	A	B	B	A-B
14	C30	B1	B	J	J	B1-J
15	C33	C1	C	C	C	C1-C
16	C34	A	A	B	B	A-B
17	C35	A	A	B	B	A-B
18	C37	A2	A	B	B	A2-B
19	C38	C	C	C	C	C-C
20	C39	A	A	B	B	A-B
21	C41	A	A	B	B	A-B
**Lake Engure (n = 19)**						
22	ENG19	D	D	B	B	D-B
23	ENG20	C	C	B	B	C-B
24	ENG21	A	A	B	B	A-B
25	ENG22	A	A	B	B	A-B
26	ENG24	A	A	B	B	A-B
27	ENG26	D	D	B	B	D-B
28	ENG28	D	D	F	F	D-F
29	ENG32	D	D	B	B	D-B
30	ENG34	C	C	F	F	C-F
31	ENG36	A	A	B	B	A-B
32	ENG39	D	D	C7	C	D-C7
33	ENG41	D	D	F	F	D-F
34	ENG42	A	A	B	B	A-B
35	ENG43	A	A	B	B	A-B
36	ENG45	A	A	L9	-	A-L9
37	ENG46	D2	D	F4	F	D2-F4
38	ENG47	A	A	A	A	A-A
39	ENG48	D	D	F	F	D-F
40	ENG49	D	D	C8	C	D-C8
**Lake Drūkšiai (n = 22)**						

41	D. E. 1	B	B	C	C	B-C
42	D. E. 2	A	A	B	B	A-B
43	D. E. 3	A	A	A	A	A-A
44	D. E. 4	C	C	C	C	C-C
45	D. E. 5	C	C	C	C	C-C
46	D. E. 6	C2	C	C	C	C2-C
47	D. E. 7	C	C	C	C	C-C
48	D. E. 8	C	C	C	C	C-C
49	D. E. 9	B	B	J	J	B-J
50	D.E. 10	A	A	B	B	A-B
51	D. E. 11	A	A	A	A	A-A
52	D. E. 12	C	C	C	C	C-C
53	D. E. 13	A	A	B	B	A-B
54	D. E. 14	A	A	A	A	A-A
55	D. E. 15	A	A	B	B	A-B
56	D. E. 16	A	A	A	A	A-A
57	D. E. 17	C	C	C	C	C-C
58	D. E. 19	A	A	B	B	A-B
59	D. E. 20	A	A	B	B	A-B
60	D. E. 21	A	A	B	B	A-B
61	D. E. 22	A	A	B	B	A-B
62	D. E. 26	A	A	A	A	A-A
**Chernobyl area (n = 1)**						
63	UK1	C	C	C	C	C-C
**Elektrėnai Reservoir (n = 8)**						
64	EM3	D3	D	F	F	D3-F
65	EM4	D	D	C	C	D-C
66	EM5	A	A	B	B	A-B
67	EM12	A	A	A	A	A-A
68	EM16	A	A	A	A	A-A
69	EM17	D	D	C	C	D-C
70	EM18	A	A	B	B	A-B
71	EM19	A	A	B	B	A-B
**GenBank (n = 7)**						
72	AP005995 (China)	New1[b]	-	C	C	New1-C
73	MZ461595 (China)	New1[b]	-	C	C	New1-C
74	CM020933 (France)	D	D	F	F	D-F
75	MT410943 (Denmark)	D	D	F	F	D-F
76	KM410088 (Hungary)	New2[c]	-	M	M	New2-M
77	LC495488 (Denmark)	D	D	F[d]	F	D-F[d]
78	AP018422 (Denmark)	D	D	F[d]	F	D-F[d]

[a] without 1 bp deletion this could be ascribed as haplotype B of D-loop, [b] new *ATP6* region haplotype detected in China, [c] new *ATP6* region haplotype detected in samples containing D-loop haplotype M, [d] new D-loop haplotype from haplogroup F possessing 1 bp deletion.

Supplementary Table S3. Comparison of overall mtDNA *ATP6* and D-loop region haplotypes within studied macrogeographic area. Black, red and green letters represent unique haplotypes, i.e. found only in one of the studied countries, haplotypes detected in all studied countries and haplotypes detected only in Latvia and Lithuania, respectively.

	**Haplotypes**
	***ATP6* region**
**Latvia (n = 40)**	**A (22), A2 (1), B (1), B1 (1), C (4), C1 (1), D (9), D2 (1)**
**Lithuania (n = 108)**	**A (56), A1 (1), A3 (1), B (4), C (24), C2 (1), D (19), D3 (2)**
**Belarus (n = 37)**	**A (5), B (19), C (13)**
**Ukraine (n = 8)**	**A (1), B (3), C (3), D1 (1)**
	**D-loop region**
**Latvia (n = 178[a])**	**A (11), B (73), B4 (5), B8, B9 (2), B10, B11, B12, B13, C (33), C6, C7, C8 (2), D (6), F (21), F4 (4), F7, F9, H1, J (5), L1, L2, L5 (2), L7, L9**
**Lithuania (n = 267[a])**	**A (37), A2, A6, A8 (2), B (64), B4 (3), B5, B6 (5), B7, B8, C (53), C5, C10, D (4), D1, E (2), E1, F (75), F8 (2), F10, H (5), J (2), J3, L4, L6**
**Belarus (n = 44)**	**A (5), C (35), C1, C4, F, J**

[a] some haplotypes are heteroplasmic length variants (marked L).
